# Advanced Evolution of Pathogenesis Concepts in Cardiomyopathies

**DOI:** 10.3390/jcm8040520

**Published:** 2019-04-16

**Authors:** Chia-Jung Li, Chien-Sheng Chen, Giou-Teng Yiang, Andy Po-Yi Tsai, Wan-Ting Liao, Meng-Yu Wu

**Affiliations:** 1 Department of Obstetrics and Gynecology, Kaohsiung Veterans General Hospital, Kaohsiung 813, Taiwan; nigel6761@gmail.com; 2 Department of Emergency Medicine, Taipei Tzu Chi Hospital, Buddhist Tzu Chi Medical Foundation, New Taipei 231, Taiwan; holeyeye@yahoo.com.tw (C.-S.C.); gtyiang@gmail.com (G.-T.Y.); 3 Department of Emergency Medicine, School of Medicine, Tzu Chi University, Hualien 970, Taiwan; 4 Department of Medical Research, Buddhist Tzu Chi General Hospital, Hualien 970, Taiwan; tandy@iu.edu; 5 Institute of Medicine, Chung Shan Medical University, Taichung 402, Taiwan; 6 Chinese Medicine Department, Show Chwan Memorial Hospital, Changhua 500, Taiwan

**Keywords:** cardiomyopathy, genetic mutations, cardiac remodeling, apoptosis, fibrosis

## Abstract

Cardiomyopathy is a group of heterogeneous cardiac diseases that impair systolic and diastolic function, and can induce chronic heart failure and sudden cardiac death. Cardiomyopathy is prevalent in the general population, with high morbidity and mortality rates, and contributes to nearly 20% of sudden cardiac deaths in younger individuals. Genetic mutations associated with cardiomyopathy play a key role in disease formation, especially the mutation of sarcomere encoding genes and ATP kinase genes, such as titin, lamin A/C, myosin heavy chain 7, and troponin T1. Pathogenesis of cardiomyopathy occurs by multiple complex steps involving several pathways, including the Ras-Raf-mitogen-activated protein kinase-extracellular signal-activated kinase pathway, G-protein signaling, mechanotransduction pathway, and protein kinase B/phosphoinositide 3-kinase signaling. Excess biomechanical stress induces apoptosis signaling in cardiomyocytes, leading to cell loss, which can induce myocardial fibrosis and remodeling. The clinical features and pathophysiology of cardiomyopathy are discussed. Although several basic and clinical studies have investigated the mechanism of cardiomyopathy, the detailed pathophysiology remains unclear. This review summarizes current concepts and focuses on the molecular mechanisms of cardiomyopathy, especially in the signaling from mutation to clinical phenotype, with the aim of informing the development of therapeutic interventions.

## 1. Introduction

Cardiomyopathy refers to a group of heterogeneous genetic or idiopathic cardiac diseases that feature myocardial structural and functional abnormalities that occur due to myocyte injuries [1]. The anatomic changes and cell death induce electrical dysfunction contraction abnormalities of the heart, causing arrhythmia and heart failure. The reported etiologies include chronic hypertension, valvular heart disease, and toxin exposure [2]. Ischemic heart diseases and induced myocardial disorders are also included. Genetic mitochondrial dysfunction has been a focus of study [3]. However, clinical and basic studies carried out over a long time have not clarified details of the pathophysiology. Recent studies have focused on the molecular and genetic aspects of cardiomyopathy, including local inflammation, reperfusion injury, death, remodeling, and recovery of myocardia, with the goal of informing novel approaches for clinical and prognostic assessments [4,5]. In this review, we summarize the current concepts concerning the cell biology and molecular regulation of cardiomyopathy with the aim of informing the development of therapeutic interventions.

## 2. Clinical Features of Genetic Cardiomyopathy

**Figure 1:**
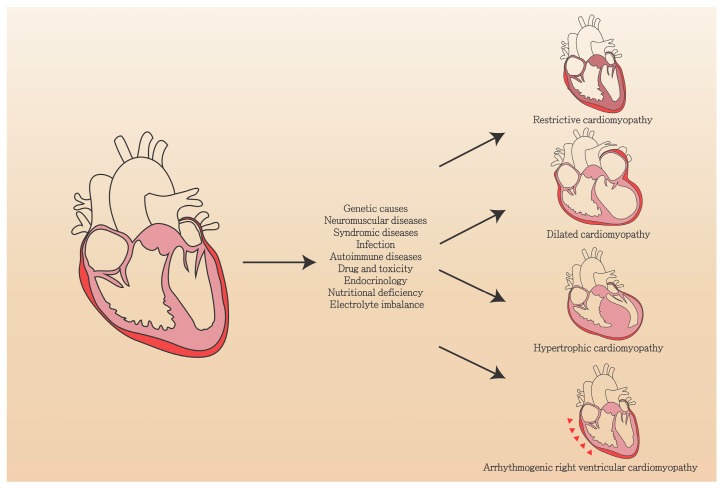
Risk factors of the four major types of cardiomyopathy.

The heterogeneous conditions that encompass cardiomyopathy feature myocardial dysfunction with structural and functional abnormalities, and are broadly classified as familial types and non-familial types [6]. Based on structural and functional abnormalities, these types can be further classified into four major categories: dilated cardiomyopathy (DCM), hypertrophic cardiomyopathy (HCM), restrictive cardiomyopathy (RCM), and arrhythmogenic right ventricular cardiomyopathy/dysplasia (ARVC/D) (Figure 1). In familial types of cardiomyopathy, left ventricular non-compaction (LVNC) is considered an unclassified type. Takotsubo cardiomyopathy and tachycardiomyopathy are two common cardiomyopathies that are classified as non-familial types [7]. The primary causes of the different types of cardiomyopathies are heterogeneous, but they share a final common pathway leading to cardiac dysfunction.

## 3. DCM

DCM is characterized by the structural dilation of ventricles associated with poor contraction. DCM is the most common cardiomyopathy, with an incidence ranging from 5–8 cases per 100,000 people [8]. Similar to the other three major types of cardiomyopathies, DCM is classified as primary or secondary types. In primary DCM, genetic causes that are independent of age include Titin (*TTN*), Lamin A/C (*LMNA*), Myosin heavy chain 7 (*MYH7*), and Troponin T2 (*TNNT2*) mutations. The familial DCM accounts for 20–48% of total DCM [9]. Mutations that affect sarcomeric and intrasarcomeric proteins can induce decreased contraction force via calcium signaling in mechanotransduction pathways [10]. Mutation of *LMNA*, which encodes nuclear lamin A and nuclear lamin C, is important in the pathogenesis of DCM. *LMNA*-related DCM may present with left ventricular enlargement and poor systolic function, promoting significant conduction system dysfunction and arrhythmias [11,12]. Peters et al. [13] reported 11 genes associated with DCM and ventricular arrhythmias, including *LMNA,* sodium voltage-gated channel alpha subunit 5 *(SCN5A),* RNA binding motif protein 20 *(RBM20),* filamin C *(FLNC)*, and *TTN*. In these populations, in which the mortality rate can be high, the implantable cardioverter-defibrillator (ICD) may provide benefit for the primary prevention of sudden cardiac death. 

Etiologies reported in secondary DCM include myocarditis, Kawasaki disease, Churg-Strauss syndrome, drug-related toxicity, endocrine disturbance, hypophosphatemia, hypocalcemia, and tachycardiomyopathy [6,14,15]. Dilated cardiomyopathy from ischemic cardiomyopathy is an important etiology. Patients with ischemia-induced DCM may present with left ventricular dysfunction and wall motion hypokinesia. Iskandrian et al. [16] described the assessment of right ventricular function to rule out ischemic cardiomyopathy, based on the knowledge that preserved right ventricular function is a characteristic in ischemia-induced DCM. The detailed mechanism has been investigated. Ischemic events may trigger the accumulation of oxidative stress and promote apoptosis of cardiomyocytes [17]. After percutaneous coronary intervention, the enhanced production of reactive oxygen species from reperfusion injury also causes cell death, which induces cardiac remodeling to form DCM. Early diagnosis and timely intervention of ischemic events are effective to prevent DCM. 

Pediatric DCM is a rare disorder, with 0.57 cases per 100,000 person-years reported in the United States [18]. When it occurs, it is serious. Risk factors include gender (0.66 vs 0.47 cases per 100,000 in boys vs. girls, respectively), race (0.98 vs 0.46 cases per 100,000 in blacks vs. whites, respectively), and age (4.40 vs 0.34 cases per 100,000 in infants < 1 year-of-age vs. children, respectively) [18]. Common causes of pediatric DCM are myocarditis (46% of cases) and neuromuscular disease (26% of cases). The clinical outcome is poor, with reported 1-year and 5-year rates of death or heart transplantation of 31% and 46%, respectively [19]. Similar genetic etiologies are suspected in adult and pediatric DCM. However, the medications routinely used in adult DCM are not effective in pediatric patients. Patel et al. [20] reported that pediatric and adult DCM are induced by different pathophysiological conditions and that pediatric DCM does not feature adverse remodeling [20]. The detailed pathophysiology in pediatric DCM is unclear. 

Secondary etiologies of cardiomyopathy include infection, autoimmune diseases, drug-related toxicity, endocrinology, nutritional deficiency, and electrolyte imbalance. These can also impair the contraction force and decrease transmission [21]. To compensate for left ventricular (LV) systolic dysfunction, the LV remodeling process produces chamber dilatation to form DCM, due to an inappropriate transcriptional response to biomechanical stress [22,23]. The detailed etiology of DCM is summarized in Table 1. 

Clinical symptoms, including orthopenia, dyspnea, or pitting edema, are present in 80% of DCM patients [8]. Other associated symptoms that can appear in advanced DCM cases include abdominal distention, nausea, poor appetite, fatigue, and cachexia. The initial physical examination typically reveals bilateral peripheral pitting edema, tachycardia, engorged jugular vein, and bilateral crackle. The structural and electrical dysfunctions result in abnormal rhythms in an electrocardiogram (ECG), which include non-specific repolarization abnormalities, LV hypertrophy, pathological Q-waves, poor R wave progression, prolonged PR interval (measured from the beginning of the P wave to the beginning of the QRS complex), atrioventricular block, or left bundle branch block. Global LV hypokinesis and dilation are typically detected by echocardiography. In chronic DCM cases, intraventrical thrombi and mitral regurgitation may be detected due to annular dilatation. In one study, 1-year mortality ranged from 25–30% and the 5-year mortality was 50% [24]. LV ejection fraction < 25%, right ventricular (RV) dilation, advanced heart failure class, and poor hemodynamic status at cardiac catheterization are poor prognosis factors related to the development of pulmonary hypertension and mortality [6,21].

Genetic mutations of DCM are important in progressive heart failure, and account for approximately 50% of cases [25]. However, the familial DCM prevalence is difficult to estimate. In a meta-analysis of 23 studies, the prevalence of familial DCM was approximately 23% with a wide range of 2% to 65% [26]. Approximately 40% of cases of familial DCM have an identifiable genetic cause [27]. The mutations involve genes encoding diverse proteins, including those associated with the sarcomere, cytoskeleton, sarcolemma, ion channels, and intercellular junctions. The mutations impair cellular structures and muscle contraction via various pathways. Mutations in sarcomere proteins are important in RCM, accounting for 30% of the total cases, and include *TTN, MYH7, MYH6, TNNT2,* tropomyosin 1 *(TPM1),* and *troponin C1 (TNNC1)*. *TTN* is the largest human protein and is a component of muscle. Mutation in the *TTN* gene is key in the potential for disease [28]. *TTN* mutation impairs associated signaling that is vital to the contraction and relaxation of striated muscle [29,30]. The dysfunction of the titin protein also induces defective transmission of force and transduction [31,32]. Mutations in the *MYH7* gene, which encodes β-myosin heavy chain, and *TNNT2*, which encodes troponin T, also lead to sarcomere unit dysfunction. Troponin T binding to troponin C/I forms the troponin complex, which regulates muscle contraction via calcium sensitivity in the interaction between actin and myosin heavy chain. In DCM, *TNNT2* mutation decreases calcium sensitivity in muscle contraction [33].

## 4. HCM

HCM is a genetically determined cardiomyopathy characterized by nondilated LV hypertrophy (LVH) [34]. Epidemiology studies have indicated a prevalence of HCM of one case per 500 people [35,36]. The primary cause of HCM are mutations in genes encoding cardiac sarcomeric proteins [37] and AMP kinase, such as *TTN, MYH7, MYH6, TNNT2, TNNI3, TPM1, MBYPC3, TNNC1, MYH7,* and *ACTC1*. Mutation of the *MBYPC3* gene, which encodes myosin binding protein C, is reportedly associated with HCM formation. In a transgenic mouse model with mutated *MYBPC3*, sarcomere dysfunction in the heart was evident and HCM formation was promoted [38,39]. The mutations decrease ATP production and regulate contraction force [40,41,42]. In HCM, these sarcomeric or adenosine triphosphate (ATP) kinase protein mutations may induce increased contraction force via calcium signaling in mechanotransduction pathways and decreased ATP production, leading to LV diastolic dysfunction [43,44,45]. Several mechanisms associated with pathogenesis include mechanotransduction, Ras-Raf-mitogen-activated protein kinase (MEK)-extracellular signal-activated kinase (ERK) pathway, protein kinase C (PKC) signaling, mothers against decapentaplegic homolog (SMAD), and mitogen-activated protein kinase (MAPK) pathways. These also promote HCM formation [4,46].

Secondary etiologies include hypertension, obesity, aortic stenosis, athletic training, and deposition of amyloid [47,48]. In genetic HCM, two major mutations in the *MYH7* and *MYBPC3* genes account for approximately 80% of the total cases [49,50]. The two genes are involved in the induction of different signaling pathways associated with the progression of HCM. The *MYH7* gene encodes the myosin heavy chain in the sarcomere thick filament proteins, which hydrolyze ATP to produce the force required for muscle contraction. Myosin typically consists of a globular head domain and coiled-coil rod domain. The globular head domain protrudes into the interfilament space to interact with thin filaments [51]. The mutation of *MYH7* involves amino acid substitutions in important residues and domains, especially in the ATPase and actin binding domains, which are important in the transmission of force [52]. The result is a dysfunction that leads to HCM. This scenario was confirmed in a transgenic rabbit model featuring a mutated *MYH7* R400Q gene and in Maine Coon Cats [53,54]. The mutation of the *MYBPC3* gene, which encodes cardiac myosin binding protein-C (cMyBP-C), usually results in the absence of the protein. In HCM, *MYBPC3* mutations encode pathological truncated proteins. These protein products are not found in HCM tissue [55]. The protein levels in symptomatic heterozygous HCM carriers are low. The results indicated that pathological HCM may be caused by the haploinsufficiency of *MYBPC3* mutations [56]. Confirmation of the mechanism was provided by the demonstration of the reduced cMyBP-C level in human symptomatic heterozygous carriers of *MYBPC3* mutations compared to normal heart tissue [57]. The mutation impairs the binding sites of myosin and titin via the carboxyl-terminus of cMybp-C, leading to cMyBP-C incorporation in the sarcomere [50,58,59]. cMyBP-C typically consists of eight immunoglobulin domains and three fibronectin domains. It protrudes into the interfilament space and interacts with actin via phosphorylation to regulate cross-bridge cycling [60,61,62,63,64]. In HCM, the mutation of cMyBP-C produces aberrant localization in sarcomeres, which induces increased tension Ca^2+^ sensitivity and decreases contraction power output [38,39].

Based on recent reports, “sarcomere-positive” mutation individuals vary from approximately 25% to 65% [65,66,67,68]. Approximately 50% of HCM patients are not hosts of sarcomeric gene mutations. The mutations in genes encoding calcium handling proteins are another subclass of genetic etiologies. Several mutations in genes encoding calcium handling proteins have been investigated. They include *TNNC1* (which encodes cardiac troponin C), *PLN* (which encodes phospholamban), and *JPH2* (which encodes junctophilin 2) [69,70,71]. Other mutations in calcium handling proteins, such as *RYR2* (which encodes ryanodine receptor 2), *CASQ2* (which encodes calsequestrin 2), *CALR3* (which encodes calreticulin 3), and *SRI* (which encodes sorcin), have varying degrees of associations to the HCM [48,72].

Biomechanical stress, cytokines, and other growth factors may trigger the Ras-Raf-MEK-ERK, PKC signaling, SMAD, and MAPK pathways, which activate nuclear transcription factors. The resulting cardiac remodeling via cell proliferation and differentiation leads to HCM [7]. ECG reveals typical LVH and other associated findings, such as large QRS complex, Q-waves, and frequent T-wave inversion. The doppler echocardiogram is a common tool to assess HCM. The obstruction of LV outflow tract with a gradient >30 mmHg is reported in approximately 25% of HCM patients [73,74]. Other findings in HCM include normal or reduced LV volume, diastolic dysfunction, and increased systolic pressure gradients. Progressive HCM may induce cardiovascular events, including sudden death due to arrhythmia, chronic heart failure, and atrial fibrillation with stroke. In advanced HCM, the placement of an ICD is an important means of preventing sudden death caused by arrhythmia. Other effective treatments for symptomatic HCM include surgical myectomy and alcohol septal ablation, controlling the symptoms of heart failure, and progressive atrial fibrillation. In end-stage HCM, heart transplantation is necessary.

## 5. RCM

RCM is a lethal cardiomyopathy characterized by LV diastolic dysfunction without LV dilation. The etiology of RCM includes genetic initiators, skeletal muscle myopathies, local scarring, infection, and deposition of amyloid [75]. In familial RCM, sarcomeric and cytoskeletal gene mutations commonly reported in RCM include *TNNI3, TNNT2, MYH7, DES, MYBPC3, LMNA, FLNC,* and *LAMP2*. These mutations are involved in the progression of RCM due to impaired actin-myosin interactions and cardiac contractility [76,77,78]. The sarcomere typically consists of two major components, actin and myosin, which comprise the thin and thick filament. The filaments regulate muscle contraction and force development in a Ca^2+^-dependent manner via the troponin complex. Cardiac troponin consists of three subunits and is located in thin filaments. The protein regulates muscle contraction in a Ca^2+^ sensitive manner [79]. The *TNNT2* and *TNNI3* genes encode cardiac troponin I and T, respectively, which respectively function as a Ca^2+^ inhibitory subunit and Ca^2+^-binding signaling transmitter [80]. During electrical depolarization, the calcium channel allows Ca^2+^ influx and binding to cardiac troponin, which activates the troponin complex, inhibits cTnI, and leads to an interaction between actin and myosin. Nine recently-reported mutations in genes encoding cTnI markedly increase myofilament Ca^2+^ sensitivity [81,82]. The *MYH7* and *MYBPC3* gene mutations have been identified in RCM and HCM. The mutations in the actin-myosin contractile apparatus induce diastolic dysfunction and promote the progression of RCM. The list of RCM-associated mutations is often similar to those genes in HCM and DCM. However, compared to HCM and DCM, the RCM has a low rate of detection for mutations (<30%) [77,83]. In DCM, a genetic study reported a 30–40% rate of genetic mutation in familial DCM population [84]. In HCM, 50–60% of people with a family history of HCM will have a mutation identified in one of the sarcomeric genes [36,85].

Mutations in sarcomeric protein disrupt the mechanical property and the ability of muscle to contract and relax via calcium signaling in mechanotransduction pathways [86,87]. In non-familial RCM, the deposition of amyloid, scleroderma, endomyocardial fibrosis, hyper eosinophilic syndrome, and drug toxicity may promote local inflammation and biomechanical stress to trigger the pathways of Wnt signaling, protein kinase B/phosphoinositide 3-kinase (AKT/PI3K) signaling, Ras-Raf-MEK-ERK, PKC signaling, SMAD, and MAPK [88,89]. These signaling pathways involve cardiac remodeling and trigger ventricular wall stiffness, cardiomyocytes apoptosis, myofibrillar disarray, and fibrosis. Typical symptoms include pulmonary congestion, dyspnea on exertion, and syncope. Stiffness of the myocardium leads to increased ventricular pressure and typically normal ventricular dimensions, dilated atria due to systemic venous congestion, diastolic dysfunction, and normal systolic function in echocardiography [78,90]. Symptomatic RCM can be effectively treated by restricting water and salt restriction with diuretics and aldosterone antagonists. In severe cases of RCM, chelation therapy, phlebotomy, placement of an (ICD), or cardiac transplantation is considered.

## 6. ARVC (Arrhythmogenic Right Ventricular Cardiomyopathy)

ARVC (arrhythmogenic RV dysplasia) is characterized by structural change in the myocardium of RV inflow, outflow tract, or apex with fibrofatty tissue. ARVC leads to regional wall motion abnormalities, and global or RV dilation. The reported prevalence is 1 in 5000 individuals [91]. In ARVC, genetic mutations account for approximately 40–60% of cases. The mutations include genes encoding junction plakoglobin (*JUP*), desmoplakin (*DSP*), plakophilin-2 (*PKP2*), desmoglein (*DSG*), and desmocollin (*DSC*). Clinical genetic studies have revealed that 30% of cases of ARVC are caused by genetic mutation. The autosomal dominant form that is common in ARVC includes five major genes: *DSP*, *PKP2, DSG2*, *DSC2*, and *TMEM43*. The autosomal recessive form of ARVC is less common, and involves mutations of the plakoglobin and desmoplakin genes (Naxos disease and Carvajal syndrome) [92,93,94]. Desmosomes consist of five proteins: JUP, PKP2, DSP, DSG2, and DSC2. Desmosomes are responsible for cell adhesion and interaction between myocardial cells [95]. In desmosomes, desmoglein and plakophilin are located in the transmembrane surface and interact with desmocolin and plakoglobin [96]. Desmosomes are important for electrical conduction and mechanical contraction in myocytes [97]. In ARVC, mutations that affect desmosomes lead to decreased force and mechanical stress between cells due to myocyte detachment and cell death [98]. Myocyte death promotes local inflammation and fibrofatty change. The structural change induces heart remodeling by replacement of injured myocytes [99]. The scar tissue also disrupts electrical transduction and promotes the progression of ARVC. In addition, mutation of the ryanodine receptor *2 (RYR2)* gene plays an important role in ARVC [100]. Mutated *RYR2* impairs calcium release from the sarcoplasmic reticulum. The imbalance of excitation–contraction coupling due to impaired intracellular calcium content may induce arrhythmias. Intracellular calcium content change may trigger cellular death and cause cardiac fibrosis [92].

The mutations can induce arrhythmia and pathological structural change [93,101,102,103]. Cardiac-specific mutations of the desmosomal protein desmoplakin lead to nuclear localization of the desmosomal protein plakoglobin and also decrease the activation of Wnt/beta-catenin (CTNNB1) signaling mediated by T-cell factor/lymphoid enhancer-binding factor (TCF/LEF) transcription factors [104]. These signaling disturbances trigger the expression of adipogenic and fibrogenic genes, and promote ventricular dilation and fibrofatty infiltration. Prolonged ventricular fibrosis and cardiomyocyte apoptosis in cardiac remodeling can cause arrhythmia and pathological structural change that results in RV cardiomyopathy. Secondary etiologies can be triggered by the inflammatory response and lead to cardiac remodeling. Arrhythmia may be induced during athletic activity by the exercise-induced release of catecholamine, leading to sudden cardiac arrest. Other typical symptoms include palpitations, syncope, atypical chest pain, and dyspnea [105]. In ECG analysis, sustained or non-sustained monomorphic VT and left bundle branch block are common in ARVC cases [106]. Prevention of sudden cardiac death is important in ARVC patients. In advanced ARVC cases, placement of an ICD is necessary.

## 7. LVNC

LVNC was an unclassified cardiomyopathy until it was proposed as a familial type in 2008 [107]. Two features in LVNC have been reported. One is the prominent trabeculae and deep intertrabecular recesses in the LV, which form non-compacted and compacted layers of myocardium. The other feature is the continuity of the LV cavity with deep intratrabecular recesses [108]. Several hypotheses proposed to explain the pathophysiology include intrauterine arrest leading to congenital malformation of the heart [109]. Cardiac remodeling can lead to prominent trabeculations during adult life in response to the LV loading [110]. Several gene mutations have been investigated in LVNC (Table 2). The inheritance of LVNC can be common in X-linked recessive or autosomal dominant conditions, especially in Barth syndrome, resulting from mutation of the *TAZ* gene. Autosomal dominant LVNC may present with differing severities in family members, and can include septal defects and Ebstein’s anomaly [111,112,113,114]. The genetic causes are heterogeneous, but the final common signaling is similar to other cardiomyopathies [108]. A large group of sarcomere encoding gene mutations, including *LDB3, MYH7*, *ACTC1*, *TNNT2*, *MYBPC3*, *TPM1*, and *TNNI3*, have been noted in up to 20% of LVNC cases, especially in *MYH7* and *MYBPC3* mutations, with rates of up to 13% and 8% [110,115,116]. *TAZ* and *LMNA* mutations in LVNC impair calcium handling [115]. In addition, mitochondrial genome mutations and chromosomal abnormalities have also been reported in LVNC [117,118]. Several signaling pathways that have been implicated include the Wnt/planar cell polarity (PCP) and Notch signaling pathways. In the NOTCH pathway, FK506-binding protein 1A (FKBP1A), which is a novel negative modulator of activated NOTCH1 that also interacts with several intracellular protein complexes, is important in regulating cardiomyocyte proliferation [119,120]. In vitro, deficient FKBP1A expression results in ventricular hypertrabeculation and non-compaction of the ventricular wall [121]. The Wnt/PCP pathway regulates embryonic patterning and organogenesis. In the Wnt/PCP signaling pathway, Wnt binding with the frizzled receptor activates phospholipase C (PLC) to promote downstream protein activation, including inositol 1,4,5-triphosphate (IP3), 1,2 diacylglycerol (DAG), and PKC, which in turn trigger the release of calcium ions into the cytoplasm. The activated PKC can also interact with other signaling pathways, such as MAPK signaling pathways. Wnt/PCP regulates cardiomyocyte polarity, myofibrillogenesis, and development of ventricular non-compaction via a large number of PCP effectors, which include disheveled-associated activator of morphogenesis 1 (Daam1). In vitro, mutations in Wnt/PCP signaling key components, such as VANGL planar cell polarity protein 2 (VANGL2), scribble planar cell polarity protein (SCRIB), disheveled, and disheveled associated activator of morphogenesis 1 (DAAM1), have been related to the development of ventricular non-compaction [122,123,124,125,126].

## 8. Genetic Mutations in Cardiomyopathy

Genetic mutations in cardiomyopathy may lead to progressive heart failure. In children and young adults, genetic mutation is a major indication for early heart transplantation or ICD insertion [127]. Several gene mutations are valuable for timely screening in heart failure patients and for prenatal testing [16,128]. The identification of these mutations can be critical in preventing morbidity and mortality, alerting patients and their families, and for population-based studies. Understanding the mutations that are important in cardiomyopathy is essential in personalizing therapy [129]. The identified gene mutations in the five major cardiomyopathies are listed in Table 2.

## 9. Pathophysiology of Cardiomyopathy

Cardiomyopathy features multiple steps and usually involves several pathways, which include cardiac hypertrophy, G-protein pathway, fibrosis, apoptosis, and AKT signaling. Infection, metabolic disease, presence of toxins, or deposition of amyloid can lead to myocardial injury and remodeling (Figure 1). Specific mutations of these genes impair the muscular contraction mechanism due to the sensitivity of ion channels, calcium regulation, and the transmission of mechanical force. To understand the detailed mechanisms, many studies have focused on the genetic background of cardiomyopathy and have attempted to understand the detailed signaling pathways within the enormous complexity of cardiomyopathy. Several major pathways of cardiomyopathy have been reported and investigated. In the following subsections, we discuss the major signaling pathways that play an important role in cardiomyopathy.

### 9.1. Ras-Raf-MEK-ERK Pathway in Cardiac Myocyte Hypertrophy

**Figure 2:**
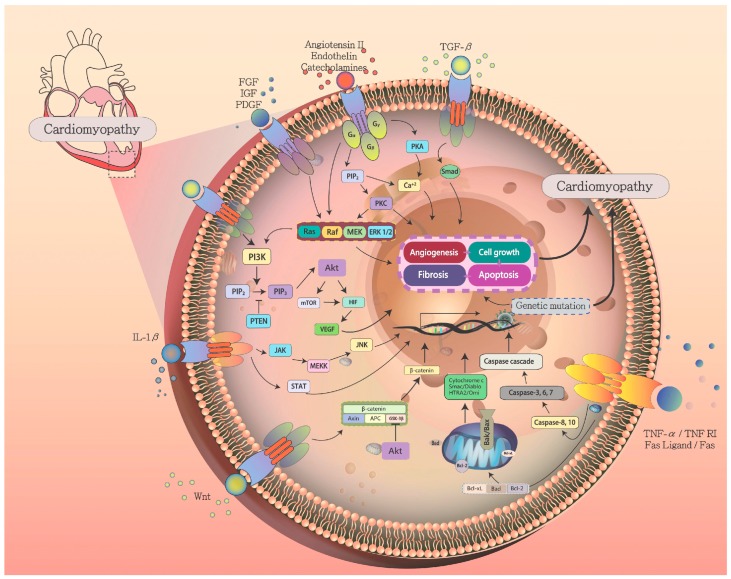
Schematic drawing of the details of the signaling pathways of five major cardiomyopathies. Several pathways, including genetic mutation, Ras/Raf/MEK/ERK signaling pathway, G-protein signaling, Wnt/β-catenin signaling pathway, AKT/PI3K Pathway, TGF-β signaling, JNK/p38 pathway, and apoptosis pathway, are involved in cardiomyopathy to promote angiogenesis, cell growth, apoptosis and fibrosis, leading to ventricular dilation, hypertrophy, or restriction. FGF: Fibroblast growth factor; IGF: Insulin like growth factor; PDGF: Platelet derived growth factor; TGF: transforming growth factor; PKA: Protein kinase A; PIP2: Phosphatidylinositol 4,5-bisphosphate; PKC: Protein kinase C; MEK: Mitogen-activated protein kinase kinase; ERK: Extracellular-signal-regulated kinase; AKT: Protein kinase B; IL: Interleukin; PTEN: Phosphatase and tensin homolog, PI3K: Phosphoinositide 3-kinases; JAK: Janus kinase; MEKK: Mitogen-activated protein kinase kinase kinase; JNK: c-Jun N-terminal kinase; STAT: Signal transducer and activator of transcription; APC, TNF: Tumor necrosis factor receptor type 1; Fas: Fas cell surface death receptor.

A variety of pathologic or physiological stress stimuli can impair contractility and Ca^2+^ sensitivity. The affected cardiomyocytes adapt to the increased demands for cardiac work. The increased muscle mass and functional demand of the cardiomyocytes results in the increased thickness of the ventricular walls. This reduces the ventricular volume, which leads to decreased cardiac output. In advanced cases, the poor cardiac output may promote acute heart failure. At the cellular level, the genetic mutation and environment stresses from infection, toxin exposure, autoimmune response, or endocrine imbalance induce several signaling pathways to promote hypertrophy of cardiac myocytes and augmented contractile capacity. As an early response to hypertrophy stress, cell cycle regulation is activated to increase proliferation and inhibit apoptosis of myocytes [130]. The Ras-Raf-MEK-ERK pathway plays a central role in cardiac hypertrophy. The pathway can be activated by several neurohumoral factors, such as cytokines, growth factors, and mechanical stress. Interleukin (IL)-1β promotes the Janus kinase/signal transducers and activators of transcription (JAK-STAT) signaling pathway and regulates Ras to activate the Ras-Raf-MEK-ERK pathway. In addition, cytokines also activate downstream gp130 pathways to inhibit apoptotic pathways [131]. Growth factors from cardiac myocytes and non-myocytes, such as fibroblast growth factor, transforming growth factor-beta (TGF-β), insulin-like growth factor, and platelet-derived growth factor, induce cardiomyocyte hypertrophy [132,133]. These growth factors bind to tyrosine kinases to activate Ras. The activation of the Ras/Raf/MEK/ERK pathway promotes cell proliferation and differentiation, leading to cardiac hypertrophy (Figure 2) [134]. Gelb et al. [135,136,137] reported that Ras-Raf-MEK-ERK pathway is a key signaling for cardiomyopathy. The authors described functional *RAF1* mutation rates of up to 9% in South Indian, North Indian, and Japanese populations in childhood-onset DCM cases. The *RAF1* mutation results in ERK activation in a BRAF-dependent manner to induce heart failure. In addition, the Ras-Raf-MEK-ERK pathway also plays an important role in Noonan syndrome, a developmental disorder with cardiac dysfunction, short stature, and facial dysmorphia. Pandit et al. [138] reported that missense mutations in *RAF1* impair the activity of Ras-Raf-MEK-ERK pathway via 14-3-3 binding. The authors described that most (95%) *RAF1* mutation cases present with hypertrophic cardiomyopathy (HCM). Thus, *RAF1* mutations increase ERK activation and implicate hyperactivation of Ras signaling in HCM.

### 9.2. G-Protein Signaling in Cardiomyopathy

In response to pathologic or physiological stress stimuli, cardiomyocytes or non-myocytes can release several factors to adapt to the microenvironment. These factors include catecholamines, angiotensin II, and endothelin 1. Their release activates G-protein–coupled receptor signaling. G-proteins consist of α, β, and γ subunits. The α subunit converts guanosine-5’-triphosphate (GTP) to guanosine-5’-diphosphate (GDP) and dissociates the G_βγ_ subunits to activate other downstream signaling [139,140]. The G_α_ subunits interact with enzymes, including adenylyl cyclase and phospholipase Cβ (PLCβ), which mediate ion channel activity. The G_βγ_ subunits activate several signaling pathways, such as apoptosis and ion channel activation. The G-protein subunits activate downstream signaling cascades and interact with other signaling pathways, such as the Ras-Raf-MEK-ERK, PKC, or SMAD pathway, which promote cardiomyocyte adaptation by hypertrophy and cardiac remodeling. In addition, when the cardiac contractility is reduced, the release of Endothelin 1 (EDN1) and angiotensin II (Ang II) activation of G-protein–coupled receptor signaling also regulates contractile adaptation by increasing the release of calcium [141]. The Ras-Raf-MEK-ERK pathway is activated, which triggers downstream signaling, including extracellular signal-regulated kinases (ERK1/2), p38 MAPK, and c-Jun N-terminal kinase (JNK) signaling, to mediate cardiac growth and remodeling in biomechanical stress. The activation of ERK1/2 via G-protein-coupled receptors can mediate adaptive and maladaptive cardiomyocyte proliferation (Figure 2) [142,143]. In diabetic cardiomyopathy, ERK1/2 signaling was observed in diabetic rat hearts 1 week after the induction of diabetes, which inhibited the overexpression of the antioxidant metallothionein [144].

### 9.3. Mechanotransduction Pathways

In cardiomyocytes, several proteins participate as key mechanosensors and mechanotransducers in the mechanotransduction pathway. They regulate the response to mechanical load and cellular stress by activating structural change, signaling transduction, and functional remodeling [145]. These complex protein networks interact with the sarcomere and the extracellular matrix (ECM). This mechanotransduction apparatus transfers mechanical stress from the actin–myosin complex to the sarcolemma through the sarcomere, Z-disc, and cellular cytoskeleton [146]. The components of the costamere complex connect the sarcomere and the ECM via integrins. Activation triggered by biomechanical stress and intracellular signaling can alter the contractile properties and regulate the membrane distortion via the PI3K pathway. TTN and muscle LIM protein (MLP) are two key molecular motors for force transmission and sensation within the sarcomere [147]. The detailed mechanism of TTN-directed mechanotransduction signaling was investigated in recent cardiomyopathy studies. TTN spans from the Z-disc to the M-line in sarcomeres. The protein regulates passive stiffness and functions as a signal transducer in mechanical stress overload [148]. MLP binds to α-actinin in the Z-disc. MLP is composed of two LIM domains as a mechanosensor that can activate downstream signaling. The concept of a MLP/TTN-Cap (TCAP)/TTN complex has been proposed and studied. A defect of the MLP/TCAP/TTN complex may lead to the development of cardiomyopathy and heart failure [149]. In vivo, MLP knockout mice developed a severe dilated cardiomyopathy phenotype with hypertrophy and heart failure after birth [150]. Other in vivo analyses identified a human MLP mutation (W4R) in a dilated cardiomyopathy-associated polymorphism. The mutation led to a defect in Tcap interaction/localization, which abolished binding of TCAP to MLP [150,151]. The result was confirmed in an MLP^W4R/W4R^ knock-in mouse model [152]. TTN contains a serine/threonine protein kinase domain (TK) at the M-band. The domain interacts with autophagosomal receptor protein complexes, including neighbor of BRCA1 (*NBR1*), p62 (*SQSTM1*), and muscle-specific RING finger-2 (*MURF2*) [153]. The domain also mediates autoinhibition mechanisms, including blocking of the ATP binding site via C-terminal regulatory tail and inhibiting the catalytic base via tyrosine-170. A recent analysis demonstrated that the mutation of the TTN kinase W260R (p.Trp34072Arg) can lead to early-onset cardiomyopathy and loss of the interaction with NBR1 [154]. These data support the important role of TK in cardiac muscle function and indicate that a defective TK may cause the development of myopathies.

### 9.4. AKT/PI3K Signaling in Cardiomyopathy

The AKT/PI3K signaling pathway triggers several intracellular and extracellular signals that mediate diverse cellular biology events, such as cell metabolism, growth, proliferation, survival, and angiogenesis [155,156]. The pathway is also important in cardiac adaptation that occurs due to the regulation of protein synthesis, apoptosis, and metabolism [157]. Biomechanical stress and molecular regulators, such as growth factors and cytokines, activate cell membrane receptors to trigger the activation of PI3K and transformation. PI3K phosphorylates the AKT protein by activating phosphatidylinositol 4,5-bisphosphate, phosphatidylinositol 3,4,5-trisphosphate, and phosphoinositide-dependent kinase 1. Finally, AKT inactivates the tuberous sclerosis complex, triggering downstream mechanistic target of rapamycin (mTOR) complex 1 signaling [158]. Chronic activation of the PI3K/AKT pathway occurs in cardiomyopathy. In vitro, the chronic activation of Akt1 gene expression in mice can induce adaptive cardiac hypertrophy and dilated cardiomyopathy [159]. In the acute phase, the AKT pathway can also induce cardiac angiogenesis as an adaptation to adapt cardiac stress by the induction of myocardial expression of vascular endothelial growth factor (VEGF). However, VEGF expression is decreased in the chronic phase and during pathological remodeling. In addition, mTOR also contributes to angiogenesis via the VEGF pathway and increases angiopoietin-2 by triggering the hypoxia inducible factor-1α oxygen-sensing protein (Figure 2) [159,160,161,162]. These signals promote angiogenesis during physiological cardiac hypertrophy. The overexpression of activated glycogen synthase kinase 3 (GSK-3), an AKT signaling downstream protein, negatively regulates cardiomyocyte size, reduces heart size, and decreases hypertrophy in response to pathological stimuli [163,164,165]. In hypertensive cardiomyopathy, GSK-3 is inactivated to reflect the downstream alterations of the AKT signaling cascade [166,167].

## 10. Apoptosis Signaling in Cardiomyopathy and Heart Failure

Apoptosis signaling in ischemic and other types of cardiomyopathies contributes to cardiac remodeling and heart failure [17,168]. Cardiomyocytes are terminally differentiated via apoptosis. Several signaling pathways mediate the extrinsic and intrinsic apoptotic pathways, including AKT and adrenergic stimulation [169,170]. The persistent activation of adrenergic signaling and release of cytochrome c are significantly associated with apoptosis and fibrosis [171,172,173]. Apoptosis signaling increases the accumulation of reactive oxygen species in cardiomyocytes, affects cardiac function, and impairs systolic and diastolic functions. Studies of the extrinsic and intrinsic apoptotic pathways in cardiomyopathy have revealed that biomechanical stress overload secondary to aortic banding activates the Bcl-2 family, in turn triggering the apoptosis cascade [174,175]. Intrinsic signaling is activated by binding to BCL-2 and BCL-xL to release BAX and BAK, which leads to the loss of mitochondrial outer membrane potential, causes the release of cytochrome c, and finally promotes caspase-9/3 for apoptosis [176]. The extrinsic pathway is activated by death receptors, including Fas, tumor necrosis factor receptor and death receptors to initiate the caspase cascade [177]. Caspase-8 is activated by FAS-associated death domain, which preludes apoptosis [178]. The cleavage of Bid provides a means of cross-talk between the two apoptotic pathways [179]. In HCM and DCM, the apoptotic pathway is activated via caspase signaling (Figure 2) [180,181]. In heart failure, up-regulation of TNF-induced signaling via Fas receptors is commonly observed [182,183]. Heart biopsies in cardiomyopathy patients have revealed common cell loss reflecting the tight regulation of apoptosis for stress adaptation [184]. The increased expression of caspase-3 with a high cardiomyocyte apoptotic index has been described in hypertensive patients with chronic heart failure, indicating that apoptosis signaling is increased [185]. Persistent apoptosis in cardiomyocytes along with reduced numbers of cardiomyocytes may promote the loss of contractile mass and trigger cardiac remodeling and ventricular arrhythmias in cardiomyopathy.

## 11. Myocardial Fibrosis and Remodeling

During chronic biomechanical stress and as growth factors accumulate, cardiac remodeling can result from interstitial and perivascular fibrosis. This can lead to pathological structural change in the ventricular chambers. In cardiomyopathy patients, the increased stiffness of ventricular walls significantly impairs diastolic function. The interstitial alteration occurs through stimulation of fibroblasts, which alters the collagen framework in the myocardium. After cardiomyocyte apoptosis, cardiac fibrosis occurs with deposition of collagen types I and III. This deposition compromises the function of cardiomyocytes. The resulting severe fibrosis disrupts the relaxation and contraction events, promotes stiffening of the myocardium, and induces chronic heart failure in cardiomyopathy. Fibrosis signaling is triggered by several pathways, including the TGF-β, JNK/p38, PI3K/AKT, WNT/β-Catenin, and Ras-Raf-MEK-ERK pathways. TGF-β is a key cytokine that promotes the production of ECM proteins in fibroblasts, endothelial cells, and smooth muscle cells [186,187,188]. TGF-β involves cardiac remodeling in cardiomyopathy via SMAD-mediated and non-SMAD signaling. In canonical signaling, TGF-β induces SMAD complex for translocation into the nucleus, promoting the fibrosis process. In non-canonical signaling, TGF-β signaling induces SMAD-independent pathways, including the PI3K/AKT and MAPK pathways, NF-κB, RHO/RAC1, and CDC42. In HCM, TGF-β activates hypertrophic signals by triggering myofibroblast differentiation and promotes matrix deposition. In the pressure-overloaded heart disease, the inhibition of TGF-β may control the severity of cardiac hypertrophy and fibrosis to preserve diastolic dysfunction. In DCM, TGF-β may serve as a key signal in the ischemic heart by regulating inflammatory response for scar formation. TGF-β inhibits the release of inflammatory mediators and promotes myofibroblast differentiation, leading to matrix deposition [187]. In HCM and DCM, the deposition of collagen has been associated with the TGF-β expression level. High TGF-β expression was reported in the myocardium and serum of HCM and DCM patients [189,190]. In cardiomyopathy, TGF-β activates local inflammation to facilitate clearance of apoptotic cardiomyocytes, and promotes the remodeling of fibroblasts and endothelial cells by the secretion of ECM proteins (Figure 2) [191,192]. TGF-β also activates the Ras-Raf-MEK-ERK pathway, leading to ERK translocation and phosphorylation of several transcription factors, such as the gene for activator protein 1 (*AP1*), to produce ECM proteins [193]. TGF-β may activate the JNK/p38 pathway via the association of TNF receptor associated factor 6 (TRAF6), leading to the phosphorylation of c-JUN and activation of the *AP1* transcription factor. The activation of PI3K/AKT signaling via mTOR has been reported in cardiomyopathy, along with the increased production of collagen I. In addition, this pathway can also induce epithelial-mesenchymal transition, which directly contributes to the production of collagen and development of cardiac fibrosis. The development of excessive fibrosis in patients with cardiomyopathy leads to deficits in cardiac function [194,195]. Although several studies have reported that TGF-β expression involves in DCM and HCM, the detailed mechanisms remain unclear due to lack strong evidence of TGF-β in the remodeled heart [196].

## 12. Conclusions

Cardiomyopathy is a complex disease that is triggered by several risk factors, including genetic mutation, neuromuscular disease, infection, drugs, and toxicity. There are four major types of cardiomyopathies. Several pathways are activated by biomechanical stress. Other growth factors, such as the Ras/Raf/MEK/ERK signaling pathway, G-protein signaling, Wnt/β-catenin signaling pathway, AKT/PI3K pathway, TGF-β signaling, JNK/MAPK pathway, and the apoptosis pathway, have been implicated in cardiomyopathy. These pathways promote angiogenesis, cell growth, apoptosis, and fibrosis, leading to ventricular dilation, hypertrophy, or restriction. In addition, the PI3K/AKT pathway also induces epithelial-mesenchymal transition, directly contributing to the production of collagen and cardiac fibrosis.

## Figures and Tables

**Table 1:** Common etiologies of dilated cardiomyopathy.

Etiology	Features
Gene mutation	*LMNA, MYH7, TNNT2, RBM20, TTN, BAG3, SCN5A, FLNC, TPM1, PLN, TNNC1, TNNI3, EYA4, NEBL, NEXN, ANKRD1, CSRP3, DES, SGCD, ILK, PDLIM3, ACTC1, ABCC9, CRYAB, ACTN2, TCAP, LDB3, VCL, LAMA4, MYH6, MYBPC3, MYPN, CTF1, DEM, DNAJC19, DSC2, DSP, EMD, FHL2, FKTN, FOXD4, LAMP2, PSEN1, PSEN2, SDHA, SYNE1, SYNE2, TAZ, TCAP, TMPO, TPM1, DMD*
Neuromuscular diseases	Duchenne muscular dystrophy, Becker muscular dystrophy (Mutation in dystrophin gene)
Syndromic diseases	Mitochondrial dysfunction, Tafazzin
Infection	Virus (parvovirus B19, HPV6, HIV), bacteria, Fungus, parasite
Autoimmune diseases	Polymyositis/dermatomyositis, Churg-Strauss syndrome, Wegener’s granulomatosis, Systemic lupus erythematosus, Sarcoidosis, Giant cell myocarditis
Drug and toxicity	Ethanol, Cocaine, Amphetamines, Iron overload, Antineoplastic drugs (paclitaxel, hypomethylating agents, monoclonal antibodies, tyrosine kinase inhibitors), Psychiatric drugs (Clozapine, Olanzapine, Chlorpromazine, Risperidone, Lithium, Methylphenidate, Tricyclic antidepressants, Phenothiazines)
Endocrinology	Hypothyroidism, Hyperthyroidism, Cushing’s disease, Addison disease, Pheochromocytoma, Stress, Diabetes mellitus
Nutritional deficiency	Thiamine, Zinc, Copper, Selenium
Electrolyte imbalance	Hypocalcemia, Hypophosphatemia

LMNA: Lamin A/C; MYH7: Myosin heavy chain 7; TNNT2: Troponin T2; TTN: Titin; MYH6: Myosin heavy chain 6; DSP: desmoplakin; RBM20: RNA Binding Motif Protein 20; *BAG3*: BCL2 associated athanogene 3; *SCN5A*: Sodium voltage-gated channel alpha subunit 5; *FLNC*: Filamin C; TNNC1: Troponin C1; *PLN*: Phospholamban; *EYA4*: EYA transcriptional coactivator and phosphatase 4; *TNNI3*: Troponin I3; *TPM1*: Tropomyosin 1; *NEBL*: Nebulette; *NEXN*: Nexilin f-actin binding protein; *ANKRD1*: ankyrin repeat domain 1; *CSRP3*: Cysteine and glycine rich protein 3; *DES*: Desmin; *SGCD*: Sarcoglycan delta; *ILK*: Integrin linked kinase; *PDLIM3*: PDZ and LIM domain 3; *ACTC1*: Actin; *ABCC9*: ATP binding cassette subfamily C member 9; *CRYAB*: Crystallin alpha B; *ACTN2*: Actinin alpha 2; *TCAP*: Titin-Cap; LDB3: LIM domain binding 3; *VCL*: Vinculin; *LAMA4*: Laminin subunit alpha 4; *MYBPC3*: Myosin binding protein C3; *MYPN*: Myopalladin; *CTF1*: Cardiotrophin 1; *DNAJC19*: DnaJ heat shock protein family (Hsp40) member C19; *DSC2*: Desmocollin 2; *EMD*: Emerin; *FHL2*: Four and a half LIM domains 2; *FKTN*: Fukutin; *FOXD4*: Forkhead box D4; *LAMP2*: Lysosomal associated membrane protein 2; *PSEN1*: Presenilin 1; *PSEN2*: Presenilin 2; *SDHA*: Succinate dehydrogenase complex flavoprotein subunit A; *SYNE1*: Spectrin repeat containing nuclear envelope protein 1; *SYNE2*: Spectrin repeat containing nuclear envelope protein 2; *TMPO*: Thymopoietin; *DMD*: Dystrophin; *TAZ*: Tafazzin; HPV6: Human papillomavirus 6; HIV: Human immunodeficiency virus.

**Table 2:** Genetic mutations of five major cardiomyopathies.

Cardiomyopathy	Gene Mutation
Dilated cardiomyopathy	*LMNA, MYH7, TNNT2, RBM20, TTN, BAG3, SCN5A, FLNC, TPM1, PLN, TNNC1, TNNI3, EYA4, NEBL, NEXN, ANKRD1, CSRP3, DES, SGCD, ILK, PDLIM3, ACTC1, ABCC9, CRYAB, ACTN2, TCAP, LDB3, VCL, LAMA4, MYH6, MYBPC3, MYPN, CTF1, DEM, DNAJC19, DSC2, DSP, EMD, FHL2, FKTN, FOXD4, LAMP2, PSEN1, PSEN2, SDHA, SYNE1, SYNE2, TAZ, TCAP, TMPO*
Hypertrophic cardiomyopathy	*TTN, MYH7, MYH6, MYL2, MYL3, MYBPC3, MYLK2, TNNT2, TNNI3, TPM1, ACTC, TNNC1, LDB3, CSRP3, TCAP, VCL, ACTN2, MYOZ2, NEXN, JPH2, PLN, ANKRD1, CAV3, COX15, CRYAB, GLA, LAMP2, PRKAG2*
Restrictive cardiomyopathy	*TNNI3, TNNT2, TPN1, MYH7, DES, MYBPC3, LMNA, FLNC, LAMP2*
Arrhythmogenic right ventricular cardiomyopathy	*DSP, PKP2, DSG2, DSC2, JUP, TMEM43, CTNNA3, DES, LMNA, PLN, RYR2, TGFB3, TTN, SCN5A, ARVC3, ARVC6,*
Left ventricular non-compaction cardiomyopathy	*LDB3, DTNA, TAZ, LMNA, NKX2-5, MYH7, ACTC, TNNT2, TNN13, MYBPC3, SCN5A, SNTA1, PRDM16, TPM1, NSD1, RPS6KA3, PMP22, CASQ2, MYH6*

LMNA: Lamin A/C; MYH7: Myosin heavy chain 7; TNNT2: Troponin T2; TTN: Titin; TCAP: TTN-Cap; MYH6: Myosin heavy chain 6; DSC: desmocollin; DSP: desmoplakin; PKP2: plakophilin-2; DSG: desmoglein; JUP: junction plakoglobin; RYR2: ryanodine receptor 2; ARVC: arrhythmogenic right ventricular cardiomyopathy; CASQ2: which encodes calsequestrin 2; RBM20: RNA Binding Motif Protein 20; *BAG3*: BCL2 associated athanogene 3; *SCN5A*: Sodium voltage-gated channel alpha subunit 5; *FLNC*: Filamin C; *TPM1*: Tropomyosin 1; *PLN*: Phospholamban; *TNNC1*: Troponin C1; *TNNI3*: Troponin I3; *EYA4*: EYA transcriptional coactivator and phosphatase 4; *NEBL*: Nebulette; *NEXN*: Nexilin f-actin binding protein; *ANKRD1*: ankyrin repeat domain 1; CSRP3: Cysteine and glycine rich protein 3; *DES*: Desmin; *SGCD*: Sarcoglycan delta; *ILK*: Integrin linked kinase; *PDLIM3*: PDZ and LIM domain 3; *ACTC1*: Actin; *ABCC9*: ATP binding cassette subfamily C member 9; *CRYAB*: Crystallin alpha B; *ACTN2*: Actinin alpha 2; *LDB3*: LIM domain binding 3; *VCL*: Vinculin; *LAMA4*: Laminin subunit alpha 4; *MYBPC3*: Myosin binding protein C3; *MYPN*: Myopalladin; *CTF1*: Cardiotrophin 1; *DNAJC19*: DnaJ heat shock protein family (Hsp40) member C19; *EMD*: Emerin; *FHL2*: Four and a half LIM domains 2; *FKTN*: Fukutin; *FOXD4*: Forkhead box D4; *LAMP2*: Lysosomal associated membrane protein 2; *PSEN2*: Presenilin 2; *SDHA*: Succinate dehydrogenase complex flavoprotein subunit A; *SYNE1*: Spectrin repeat containing nuclear envelope protein 1; *SYNE2*: Spectrin repeat containing nuclear envelope protein 2; *TAZ*: Tafazzin; *TMPO*: Thymopoietin; *MYL2*: Myosin Light Chain 2; *MYL3*: Myosin Light Chain 3; *MYLK2*: Myosin light chain kinase 2; *LDB3*: LIM domain binding 3; *MYOZ2*: Myozenin 2; *JPH2*: Junctophilin 2; *CAV3*: Caveolin 3; *COX15*: Cytochrome c oxidase assembly homolog COX15; *GLA*: Galactosidase alpha; *PRKAG2*: Protein kinase AMP-activated non-catalytic subunit gamma 2; *TPN1*: Vitamin B6 transporter TPN1; *TMEM43*: transmembrane protein 43; *CTNNA3*: Catenin alpha 3; *TGFB3*: Transforming growth factor beta 3; *DTNA*: Dystrobrevin alpha; *NKX2-5*: NK2 Homeobox 5; *TNN13*: Troponin I3; *SNTA1*: Syntrophin alpha 1; *PRDM16*: PR/SET domain 16; *NSD1*: Nuclear receptor binding SET domain protein 1; *RPS6KA3*: Ribosomal protein S6 kinase A3; *PMP22*: Peripheral myelin protein 22.
